# YOLO-Pika: a lightweight improved model of YOLOv8n incorporating Fusion_Block and multi-scale fusion FPN and its application in the precise detection of plateau pikas

**DOI:** 10.3389/fpls.2025.1607492

**Published:** 2025-08-20

**Authors:** Yihao Liu, Jianyun Zhao, Changjun Xu, Yuedi Hou, Yuxiang Jiang

**Affiliations:** ^1^ College of Geological Engineering, Qinghai University, Xining, China; ^2^ Qinghai Provincial Key Laboratory of Geospatial Information Technology and Application, Department of Natural Resources of Qinghai Province, Xining, China; ^3^ College of Resource and Environmental Sciences, Wuhan University, Wuhan, China; ^4^ College of Engineering, Qinghai Institute of Technology, Xining, China

**Keywords:** unmanned aerial vehicle (UAV), pika, lightweighting, image detection, YOLO

## Abstract

The plateau pika (*Ochotona curzoniae*) is a keystone species on the Qinghai–Tibet Plateau, and its population density—typically inferred from burrow counts—requires rapid, low-cost monitoring. We propose YOLO-Pika, a lightweight detector built on YOLOv8n that integrates (1) a Fusion_Block into the backbone, leveraging high-dimensional mapping and fine-grained gating to enhance feature representation with negligible computational overhead, and (2) an MS_Fusion_FPN composed of multiple MSEI modules for multi-scale frequency-domain fusion and edge enhancement. On a plateau pika burrow dataset, YOLO-Pika increases mAP50 by 3.4 points and mAP50–95 by 5.0 points while reducing parameters by 22.7% and FLOPs by 0.01%; AP improves for small, medium, and large targets. On a public Brandt’s vole hole dataset, it achieves a further 4.9-point gain in mAP50 and reduces false detections from localization errors, redundancy, and background noise by 30–50%. Compared with five state-of-the-art lightweight detectors (including YOLOv10), YOLO-Pika attains the highest detection accuracy with the fewest parameters. These results show that YOLO-Pika balances real-time performance, detection precision, and deployment feasibility, offering a practical, scalable solution for rodent burrow detection and alpine grassland damage assessment with strong cross-regional generalization.

## 1 Introduction

Renowned as the “Asian Water Tower,” the Qinghai-Tibet Plateau contains the most extensive grassland ecosystems in the world. Qinghai Province, located in the upper reaches of the Yellow River and within the northeastern region of the Qinghai-Tibet Plateau, represents a typical transitional zone characterized by both river basin features and a cold alpine grassland ecosystem. Its grassland area constitutes approximately 27% of the total grassland area of the Qinghai-Tibet Plateau. However, a growing body of scientific evidence indicates that grassland degradation has been continuously expanding in recent years (Liu et al., 2015; Zhou et al., 2017). This trend has triggered significant ecological consequences, including shifts in species composition, reductions in biodiversity, and ultimately, a decline in ecosystem stability and the provision of essential ecological services (Gao et al., 2021; Wang et al., 2019). In the current era, to effectively implement the principle of “ecological conservation as a priority and green development as a guiding framework,” it is critically important to establish an integrated management system for grassland protection and degradation control. Addressing the challenges posed by grassland degradation and rodent infestations in a scientifically sound and practically feasible manner is therefore essential for sustaining grassland ecosystems under the present ecological context.

In prior scholarly investigations, climate change and overgrazing have been widely recognized as the principal drivers of grassland degradation (Chen et al., 2017). The plateau pika (*Ochotona curzoniae*; hereafter referred to as “pika”), a small mammalian species predominantly inhabiting the Qinghai-Tibet Plateau, has garnered increasing scientific interest due to its potential ecological significance in recent years (Smith et al., 2019). However, current research on the impact of pika activities on vegetation remains marked by significant knowledge gaps and divergent perspectives. On one hand, several studies suggest that pika burrowing and foraging behaviors may enhance soil nutrient levels (Pang et al., 2020), as well as promote vegetation productivity and species richness (Pang et al., 2021). On the other hand, alternative research perspectives argue that pika activities contribute to the acceleration of grassland degradation, leading some scholars to classify the pika as a key indicator species for degraded grassland ecosystems (Qian et al., 2021; Smith et al., 2019). The inconsistencies among existing findings, combined with limitations in methodological approaches, highlight the critical need for comprehensive and systematic investigations into the relationship between plateau pika population density and its broader ecological impacts.

The assessment of plateau pika population density is conventionally conducted by quantifying active burrow counts through field-based quadrat surveys (Chen et al., 2017). Nevertheless, this conventional methodology demonstrates significant limitations with respect to spatial representativeness and operational efficiency. Although recent advancements in remote sensing technology have enabled large-scale ecological monitoring, satellite-derived remote sensing data—such as those obtained from the Landsat series—lack sufficient spatial resolution to detect individual pika burrows accurately. This technical constraint severely hampers the broader applicability of satellite remote sensing in studies related to pika population dynamics. In recent years, unmanned aerial vehicle (UAV)-based remote sensing has emerged as a promising alternative for ecological monitoring due to its high spatial resolution, enhanced maneuverability, and field deployability (Cvijanović et al., 2025; Wu et al., 2025). Such technology offers a technically viable solution for precise and spatially extensive assessments of pika populations. However, most current UAV-based investigations of pika burrows still depend heavily on manual visual interpretation techniques. These approaches are not only resource-intensive and time-consuming but also susceptible to subjective interpretation errors and inter-observer variability.

In recent years, deep learning-based object detection methods, such as VGG (Simonyan and Zisserman, 2014), YOLO (Redmon et al., 2016), and R-FCN (Dai et al., 2016), have been increasingly applied across a wide range of academic disciplines and industrial applications. For instance (Luo et al., 2025), integrated the SE attention module into the DeepLabV3+ network architecture, significantly improving its accuracy to 0.9831 (Huang et al., 2024a) employed the CRE-YOLO model for the detection of acai trees, achieving an average precision (mAP) of 97.1% at an Intersection over Union (IoU) threshold of 0.5. Within the field of rodent burrow identification (Du et al., 2022), conducted a systematic comparison of three two-stage (Faster R-CNN, R-FCN, and Cascade R-CNN) and three one-stage (SSD, RetinaNet, and YOLOv4) deep learning-based object detection models for recognizing Brandt’s voles. Their results demonstrated that Faster R-CNN and YOLOv4 achieved the highest detection accuracy. Compared with Faster R-CNN, the YOLO framework has gained broader adoption due to its more efficient computational pipeline, which reduces inference time and lowers resource consumption (Han et al., 2024). further validated the applicability of an improved YOLOv8 model for detecting plateau pika burrows in alpine meadow ecosystems, confirming its effectiveness in this specific ecological setting. However, existing models still encounter significant challenges under complex environmental conditions, particularly in terms of limited detection accuracy and high computational demands. Moreover, plateau pika burrow detection is complicated by factors such as small target size, low color contrast relative to the background, and the heterogeneous nature of grassland ecosystems. These issues contribute to notable limitations in the reliability and robustness of current detection approaches. Therefore, there is an urgent need to develop a lightweight yet highly accurate object detection model capable of overcoming the shortcomings of conventional methods and baseline architectures. Such a model would enable efficient and precise estimation of plateau pika population density, thereby providing a solid scientific basis and technical support for ecological conservation and disaster prevention efforts in grassland ecosystems along the Qinghai section of the Yellow River Basin.

**Figure 1:**
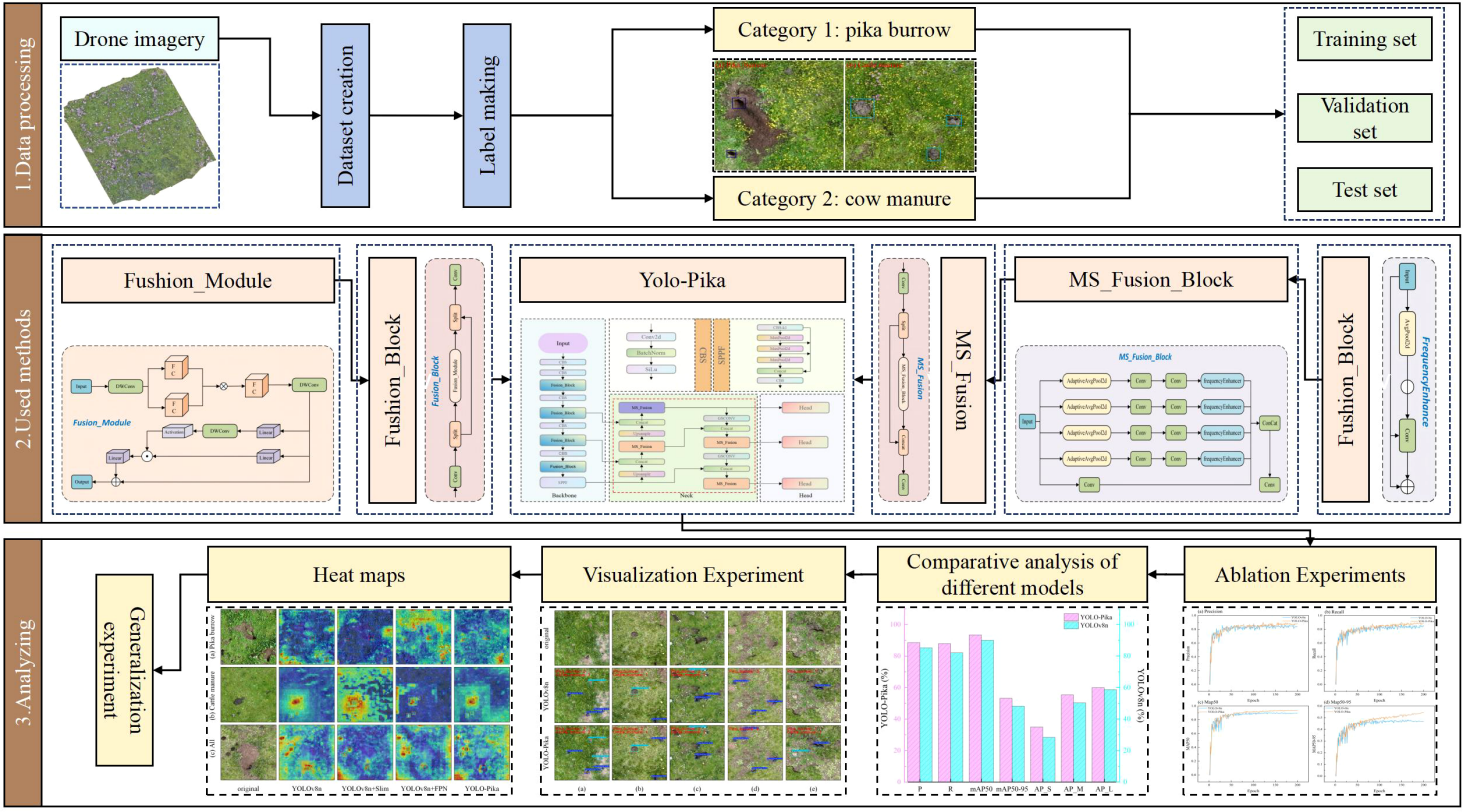
Graphical abstract.

To address the aforementioned research gaps, this paper proposes the YOLO-Pika model, which introduces the Fusion_Block module as a novel architectural component. By integrating high-dimensional nonlinear mapping with a fine-grained gating mechanism, this module enhances feature extraction capabilities and significantly improves the discriminability of small targets. Another key innovation is the introduction of the MS_Fusion module, designed to enable deep integration of multi-scale spatial features and frequency-domain information. This enhancement substantially improves the model’s ability to detect targets in complex background environments. The model further incorporates a simplified Feature Pyramid Network (FPN) structure in the neck module, which effectively reduces computational overhead while maintaining both detection accuracy and model efficiency (**Figure 1**). Compared with the conventional YOLOv8 architecture, YOLO-Pika achieves superior detection performance—both in terms of accuracy and speed—by strengthening the network’s feature extraction capability without compromising its lightweight design. This enables rapid and precise identification of plateau pika burrows. Based on an UAV remote sensing platform, the proposed model quantifies the spatial distribution density of plateau pika burrows, thereby providing data-driven support for the formulation and optimization of ecological conservation and grassland restoration strategies. The main contributions of this study are summarized as follows:

(1) The YOLO-Pika model introduces a novel Fusion_Block module that effectively integrates low-level spatial details with high-level semantic features through a combination of high-dimensional nonlinear mapping and a fine-grained gating attention mechanism. This approach significantly enhances feature representation capabilities without increasing model complexity.
(2) This study proposes a multi-scale fusion (MS_Fusion) module that combines adaptive average pooling, dual convolution operations, and frequency-domain enhancement techniques. Based on this module, a new neck architecture—referred to as MS_Fusion_FPN—is developed. This architecture significantly improves the model’s capacity for multi-scale feature extraction and integration, leading to enhanced detection accuracy across targets of varying sizes, particularly small targets.

## 2 Related work

With the rapid advancement of computer technology, deep learning-based object detection models have undergone significant improvements. Among these, the YOLO (You Only Look Once) model, as a representative single-stage detection algorithm, has been widely adopted due to its ability to perform detection tasks with high efficiency and excellent accuracy. Moreover, the YOLO model demonstrates strong reliability and ease of deployment, which highlight its importance within modern object detection frameworks. These characteristics establish it as an indispensable tool in contemporary detection systems. Therefore, for the accurate and efficient estimation of plateau pika population density, the YOLO model is considered the most suitable and effective choice.

### 2.1 YOLO series networks

In 2016, researchers including Joseph Redmon (Redmon et al., 2016) proposed the YOLO algorithm, which introduced a novel approach by formulating object detection as a regression problem. Unlike traditional two-stage detection methods, YOLO divides the input image into a grid structure and performs independent regression predictions for each grid cell. The core idea of this approach is to directly predict the bounding boxes and class labels of objects through a neural network. This design significantly streamlines the detection pipeline while maintaining a low inference latency. Also in the same year, the SSD (Single Shot MultiBox Detector) network (Liu et al., 2016) was introduced, inspired by both YOLO and Faster R-CNN. This model is capable of extracting informative features from multi-scale feature maps and performs object detection through regression-based prediction. Compared with YOLO, SSD demonstrates superior performance in terms of accuracy and robustness.

In 2017, the introduction of the Feature Pyramid Network (FPN) (Lin et al., 2017a) marked a significant advancement in object detection methodologies. By constructing a pyramid-shaped hierarchical feature structure, FPN enables the network to simultaneously exploit feature information from multiple semantic levels. This mechanism significantly enhances the model’s capability in detecting small objects with improved accuracy and efficiency. In the same year, Joseph Redmon et al (Redmon and Farhadi, 2017). proposed YOLO9000 as an extension of the original YOLO framework. The model incorporated batch normalization and anchor boxes, which effectively improved its generalization ability and overall performance. At the same time (Lin et al., 2017b), introduced the RetinaNet network model, which presented Focal Loss as a novel solution to address the persistent challenge of class imbalance between positive and negative samples in object detection. Furthermore, RetinaNet integrated the FPN architecture, enhancing its multi-scale feature extraction capability. Consequently, RetinaNet demonstrated substantial improvements in handling both class imbalance and multi-scale object detection tasks.

In 2018, Joseph Redmon proposed YOLOv3 (Redmon and Farhadi, 2018) as an extension of YOLOv2. The model adopted the Darknet-53 backbone and incorporated multi-label classification, which significantly improved detection accuracy. In 2020 (Bochkovskiy et al., 2020), introduced YOLOv4, an enhanced version built upon YOLOv3. By integrating a series of advanced techniques—including data augmentation, label smoothing (Müller et al., 2019), CIoU loss (Zheng et al., 2021), Spatial Pyramid Pooling (SPP) (He et al., 2015), Cross-Stage Partial Network (CSPNet) (Wang et al., 2020a), and Path Aggregation Network (PaNet) (Liu et al., 2018)—YOLOv4 achieved superior accuracy while maintaining high inference speed. Subsequently, Ultralytics released the YOLOv5 model, which has since undergone multiple iterative improvements. In the same year, Megvii Technology proposed YOLOX (Ge et al., 2021), which integrated key strategies such as decoupled heads, SimOTA label assignment, and anchor-free detection. These innovations resulted in a significant improvement in detection accuracy.

In 2022, the research team led by Alexey Bochkovskiy (Wang et al., 2023) introduced the YOLOv7 algorithm. By incorporating techniques such as efficient aggregation networks, dynamic label assignment, and reparameterized convolutions, YOLOv7 achieved further improvements in both detection accuracy and inference speed. Compared with its predecessors from YOLOv1 to YOLOv7, YOLOv8 introduced additional architectural optimizations. These enhancements resulted in significant gains in detection precision, real-time inference performance, and the capability to detect small objects within complex scenes. Although the YOLO series has continued to evolve in recent years, the most recent variants remain under active development and are subject to frequent updates. Consequently, their stability and community support have not yet matured.

In contrast, YOLOv8 has been extensively validated across diverse applications and is supported by a robust open-source ecosystem. Therefore, after a comprehensive evaluation of detection accuracy, real-time processing capability, and deployment reliability, YOLOv8 was selected as the baseline model. Further modifications were implemented to better align it with the requirements of grassland ecological monitoring and plateau pika population density assessment.

### 2.2 Feature-pyramid enhancements

To address the limitations of the original Feature Pyramid Network (FPN) in cross-layer semantic fusion and feature balancing, numerous researchers have proposed a variety of improvement strategies. PAFPN (Path Aggregation FPN) (Liu et al., 2018) extends the conventional top-down pathway by introducing an additional bottom-up “path aggregation” branch. Through sequential stacking of fusion nodes, it significantly enhances the propagation of low-level localization information into high-level semantic features. This mechanism effectively enriches high-level representations with spatially precise details from lower layers, improving overall detection performance. Libra R-CNN (Pang et al., 2019) addresses the foreground-background imbalance issue in multi-scale feature learning through a combination of global context pooling and progressive feature redistribution. Integrated with IoU-balanced sampling and Balanced L1 Loss, this framework not only alleviates data imbalance but also improves gradient optimization, leading to consistent performance gains across multiple object detection architectures. Context-Dependent Mining & Penalty-Incentive Allocation (CDM-PIA) (Xie et al., 2024) aims to improve the accuracy of rotated object detection. By explicitly modeling contextual dependencies at each feature pyramid level and incorporating a “penalty-incentive” gradient allocation strategy, CDM-PIA adapts the training process to better capture the geometric characteristics of rotated objects, resulting in a significant enhancement in detection precision. Collectively, these approaches extend the representational capacity of FPN from three key dimensions: path architecture design, feature redistribution mechanisms, and sample assignment strategies. These advancements highlight the importance of carefully engineering the neck module in lightweight object detection frameworks and provide valuable insights for the development of the MS_Fusion_FPN structure proposed in this study.

In contrast, YOLOv8 has been extensively validated across diverse applications and is supported by a robust open-source ecosystem. Therefore, after a comprehensive evaluation of detection accuracy, real-time processing capability, and deployment reliability, YOLOv8 was selected as the baseline model. Further modifications were implemented to better align it with the requirements of grassland ecological monitoring and plateau pika population density assessment.

### 2.3 The lightweight improvement of the YOLO algorithm

In recent years, continuous efforts have been made to develop lightweight variants of YOLOv8, further demonstrating its strong adaptability in applications that require both high detection accuracy and low computational cost. Jovanovic et al (Jovanovic et al., 2024) conducted passive rocket detection experiments using pruned and multi-scale enhanced versions of YOLOv8-nano/small. At an inference speed of 30 frames per second (FPS), they achieved an mAP50 of 0.865. This result confirmed the feasibility of deploying lightweight models for real-time, low-cost monitoring, while also highlighting the need for further improvements in detecting distant small targets (Khekan et al., 2024) modified the YOLOv8 architecture by reducing the depth of the backbone and incorporating an attention mechanism in the detection head. Their optimized model achieved an mAP of 0.995 on the CAUCAFall dataset, demonstrating significant performance gains (Huang et al., 2024b) proposed EDGS-YOLOv8, which improved the mAP to 97.1% on a drone countermeasure dataset while reducing the inference speed by 30 FPS (Zhao et al., 2024) introduced a modular and plug-and-play optimized variant specifically designed for maritime rescue scenarios. Compared with YOLOv8x, their method increased detection accuracy by 13.5% while reducing computational complexity by 85.6% (Xu et al., 2024) developed a lightweight version named YOLOv8-MPEB, which contained only 7.39 million parameters and occupied just 14.5 MB of storage. Despite its compact size, it achieved an mAP of 91.9% on a UAV helmet/reflective suit detection task. In industrial applications (Hao et al., 2024), integrated a Rep (representation pyramid)-based visual Transformer with YOLOv8 for fault detection in power transmission lines. Their approach improved average precision by 0.053, reduced floating-point operations per second (FLOPs) by 2.3, and increased detection speed to 114.9 FPS.

Previous studies have predominantly employed strategies such as backbone replacement, depthwise separable convolution, attention injection, multi-scale feature fusion, and pruning or quantization techniques. These methods have effectively improved model accuracy while significantly reducing model size, thereby demonstrating the transferability and robustness of lightweight YOLOv8 architectures. The findings provide valuable technical references and practical implementation examples for the development of the YOLO-Pika model proposed in this study, which integrates Fusion_Block and MS_Fusion. Furthermore, they highlight the critical importance and practical feasibility of pursuing lightweight design improvements in the field of ecological small-target detection.

## 3 Study area

**Figure 2:**
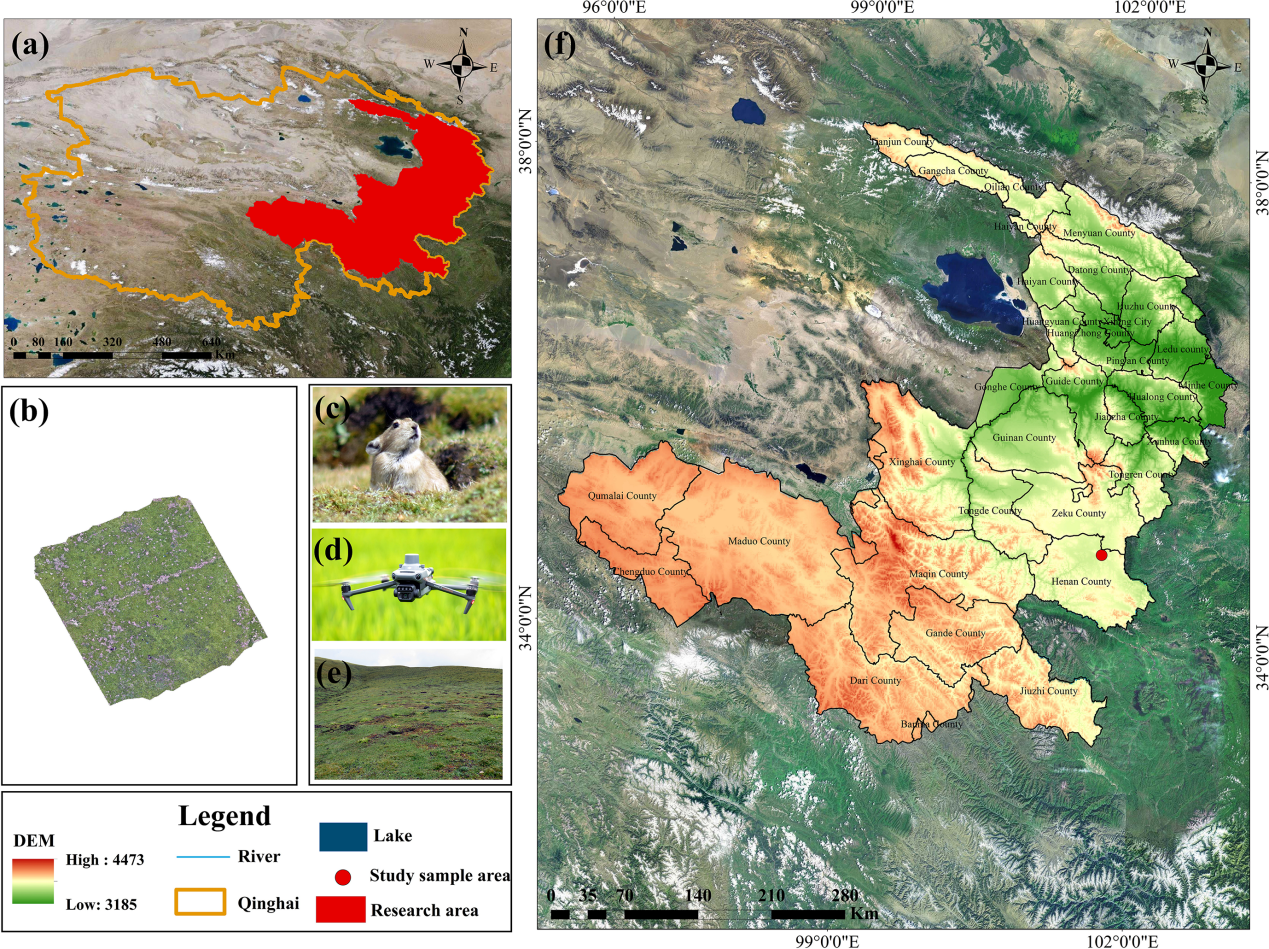
Location of the study area. **(a)** Administrative divisions of Qinghai Province; **(b)** UAV imagery; **(c)** pikas; **(d)** UAV; **(e)** Degraded grassland; **(f)** The Yellow River Basin (Qinghai section).

The study area covers the Qinghai section of the Yellow River Basin, located between 96° and 103° east longitude and 32° to 39° north latitude. With an average elevation of 4,000 meters, it lies in the northeastern part of the Qinghai-Tibet Plateau and constitutes the upper reaches of the Yellow River, specifically within the eastern region of Qinghai Province (**Figure 2a**). The Yellow River flows through several counties within this region, including Maqin, Gandê, Dari, and Jiuzhi. Zaling Lake and Eling Lake are situated in the southwestern portion of the study area. Henan County, located in the southeastern part of the Qinghai section of the Yellow River Basin (**Figure 2f**), is primarily characterized by animal husbandry and hosts the largest organic livestock production base in China. The county contains approximately 6,471.81 km² of natural grassland, representing 92.49% of its total land area. Of this, about 5,998.29 km² is classified as utilizable grassland, accounting for 92.68% of the total grassland area. Considering the high activity level of plateau pikas (**Figure 2c**) in Henan County (**Figure 2e**) (Li et al., 2019; Zhao et al., 2022) and the ongoing implementation of ecological conservation initiatives, this study selects Henan County as the representative research area within the Qinghai section of the Yellow River Basin.

## 4 Experimental conditions

### 4.1 Data set

The breeding season of the plateau pika extends from April to late June or early July (Qu et al., 2012), with the population peaking in August. By November, pikas begin to aggregate in their burrows for overwintering, leading to a marked decline in burrow activity. Notably, the lowest number of active burrows is observed during winter, which serves as the baseline for estimating population levels in the following year. Accordingly, this study conducted field surveys in August, focusing on areas where plateau pikas exhibit high activity. Aerial imagery was collected using the DJI Mavic 3E (Shenzhen DJI Technology Co., Ltd.) (**Figure 2d**), which has an effective resolution of 20 million pixels, thereby supporting high-precision data acquisition for subsequent analysis.

Flight operations and data collection were conducted daily between 8:00 a.m. and 5:00 p.m. Before initiating the formal image acquisition process, a sample plot measuring 100 m × 100 m (equivalent to 10,000 m²) was established within the plateau pika distribution area. This plot was subsequently surveyed using drone - acquired aerial imagery. A total of 11 representative sample regions exhibiting typical pika distribution patterns were examined, and high-resolution drone images were collected for each site. Additionally, considering the impact of flight altitude on the accuracy of burrow identification, this study adopted the methodology proposed by Wu et al (Wu et al., 2024). Based on their findings, the drone’s operational parameters were set as follows: a flight altitude of 15 m, a speed of 2 m/s, and waypoint and route overlap ratios of 80% and 70%, respectively (**Figure 2b**).

**Figure 3:**
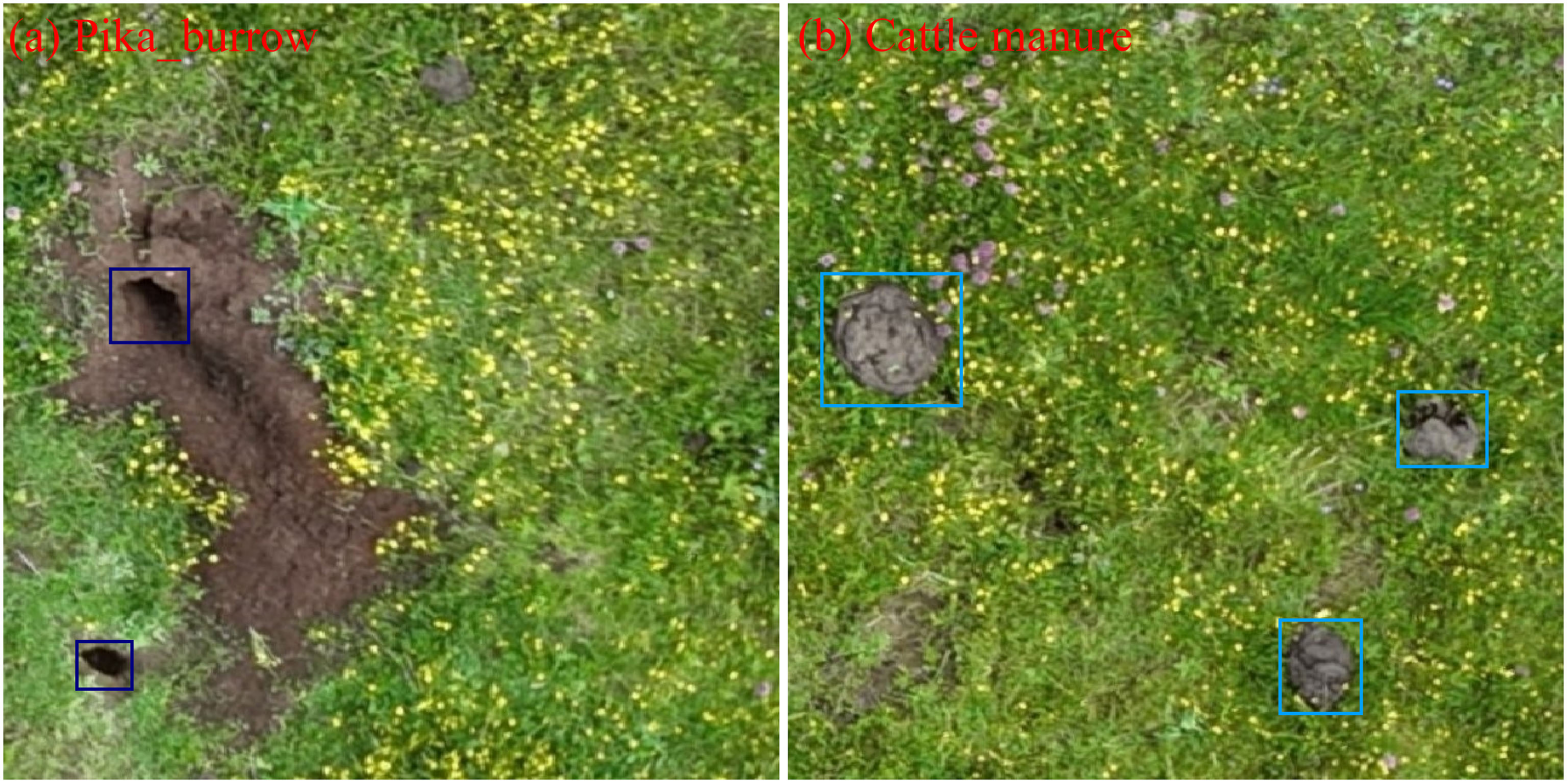
A comparative illustration of pika burrow characteristics **(a)** and cattle manure features **(b)**.

Considering factors such as shooting angles and lighting conditions, the drone-captured imagery reveals notable similarities between the visual characteristics of cow dung (**Figure 3b**) and plateau pika burrows (**Figure 3a**). To address this potential source of classification ambiguity, this study defined cow dung and pika burrows as the two primary classes within the sample dataset. The image data were batch-exported from ArcGIS Pro 3.1 for subsequent processing. Annotation was performed using the online platform Make Sense (https://www.makesense.ai/). The dataset was divided into three subsets: a training set containing 3778 images, a validation set with 472 images, and a test set comprising 474 images. The overall partitioning ratio of the dataset was established as 8:1:1, corresponding to training, validation, and testing, respectively.

### 4.2 Evaluation indicators

To ensure the robustness of the research methodology and the validity of the experimental results, this study employs mean average precision (mAP), precision (P), and recall (R) as key performance evaluation metrics (Hossin and Sulaiman, 2015). The mathematical formulations for these metrics are defined as follows:


(1) $$P=\frac{TP}{TP+FP}$$

(2) $$R=\frac{TP}{TP+FN}$$

(3) AP=∫01P(R)dR

(4) $$mAP=\frac{\sum_{i=1}^{n}AP_{i}}{n}$$

In the equation, *TP* (True Positive) indicates the quantity of positive samples that the model correctly predicts as positive. *FP* (False Positive) represents the number of negative samples that the model mistakenly predicts as positive. *FN* (False Negative) denotes the number of positive samples that the model wrongly predicts as negative.

### 4.3 Hyperparameter settings

A deep learning model was developed based on the PyTorch 1.13 framework. The computational infrastructure included an NVIDIA RTX 4060 Ti GPU with CUDA version 11.6 and an Intel Core i7-12700F CPU. During the training phase, the model was trained for a total of 200 epochs with a batch size of 32. To effectively reduce the risk of overfitting, the hyperparameter configurations detailed in **Table 1** were implemented.

**Table 1:** Hyperparameter settings.

Hyperparameter	Value
Initial learning rate (lr0)	0.01
Final OneCycleLR learning rate (lrf)	0.01
Momentum	0.937
Weight decay	0.0005
Warmup epochs	3.0

## 5 Proposed model

**Figure 4:**
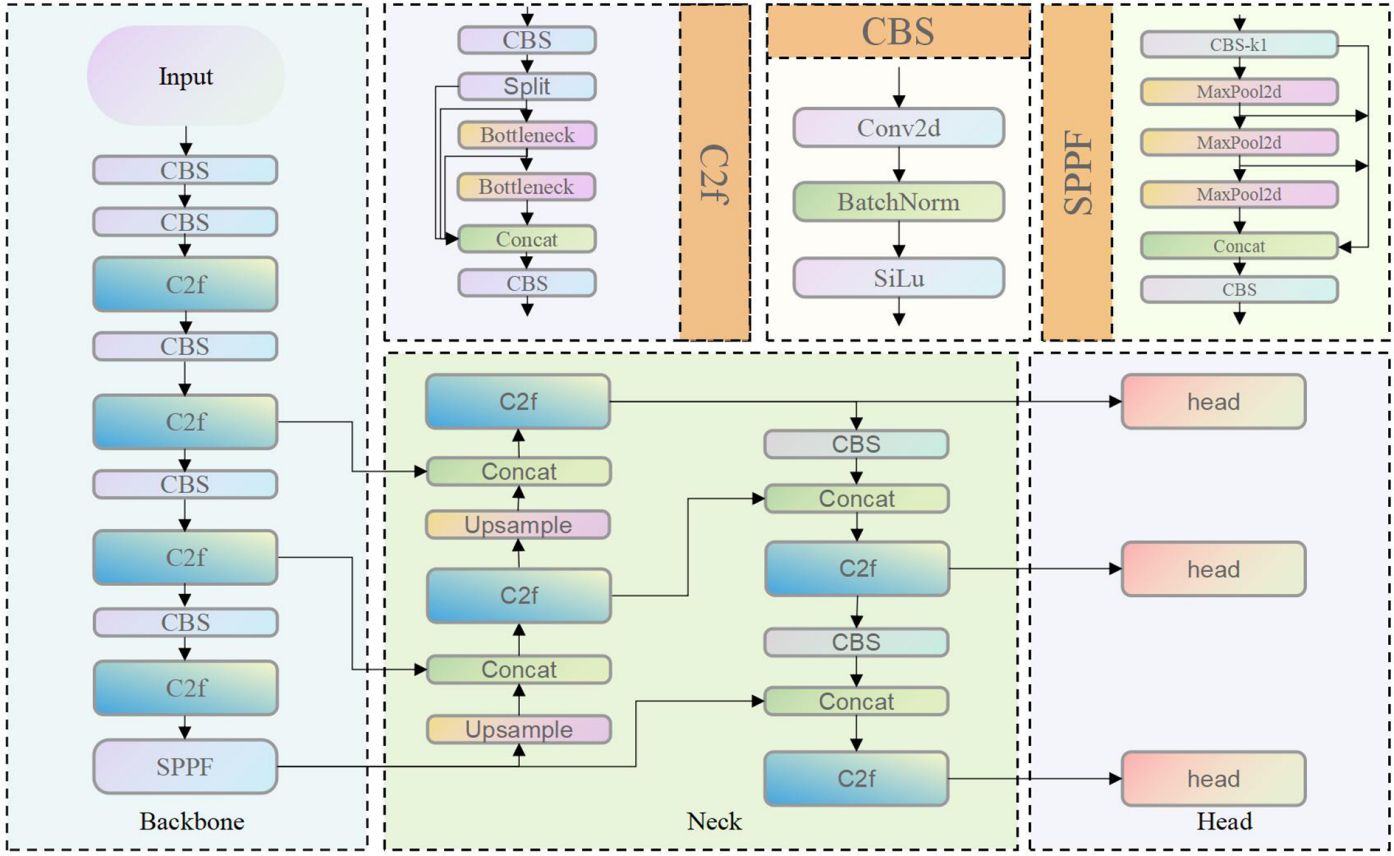
Structure of the YOLOv8.

YOLOv8 inherits the one-stage detection paradigm of the YOLO series and retains the classic architecture comprising three core components: Backbone, Neck, and Head (**Figure 4**). The model is derived from YOLOv5 through structural simplification and optimization, with the objective of improving inference efficiency and detection accuracy. Despite achieving a favorable trade-off between speed and accuracy, YOLOv8 faces challenges in capturing fine-grained local features and detecting objects with significant scale variations. Specifically, the network primarily relies on feature stacking mechanisms such as the C2f module, which provides limited enhancement in both channel-wise and spatial dimensions. Moreover, the YOLO series predominantly focuses on feature learning within the spatial and channel domains, without explicitly modeling or enhancing the high-frequency and low-frequency components of image data.

In recent years, the development of single-stage object detection models has highlighted the importance of adapting the YOLO architecture to meet specific application requirements. Accordingly, this study integrates optimization strategies from previous research and proposes a customized model architecture based on YOLOv8n for the detection of plateau pika burrows in grassland environments. This work aims to provide technical support for the effective management of grassland rodent infestations.

### 5.1 Fusion_Block

**Figure 5:**
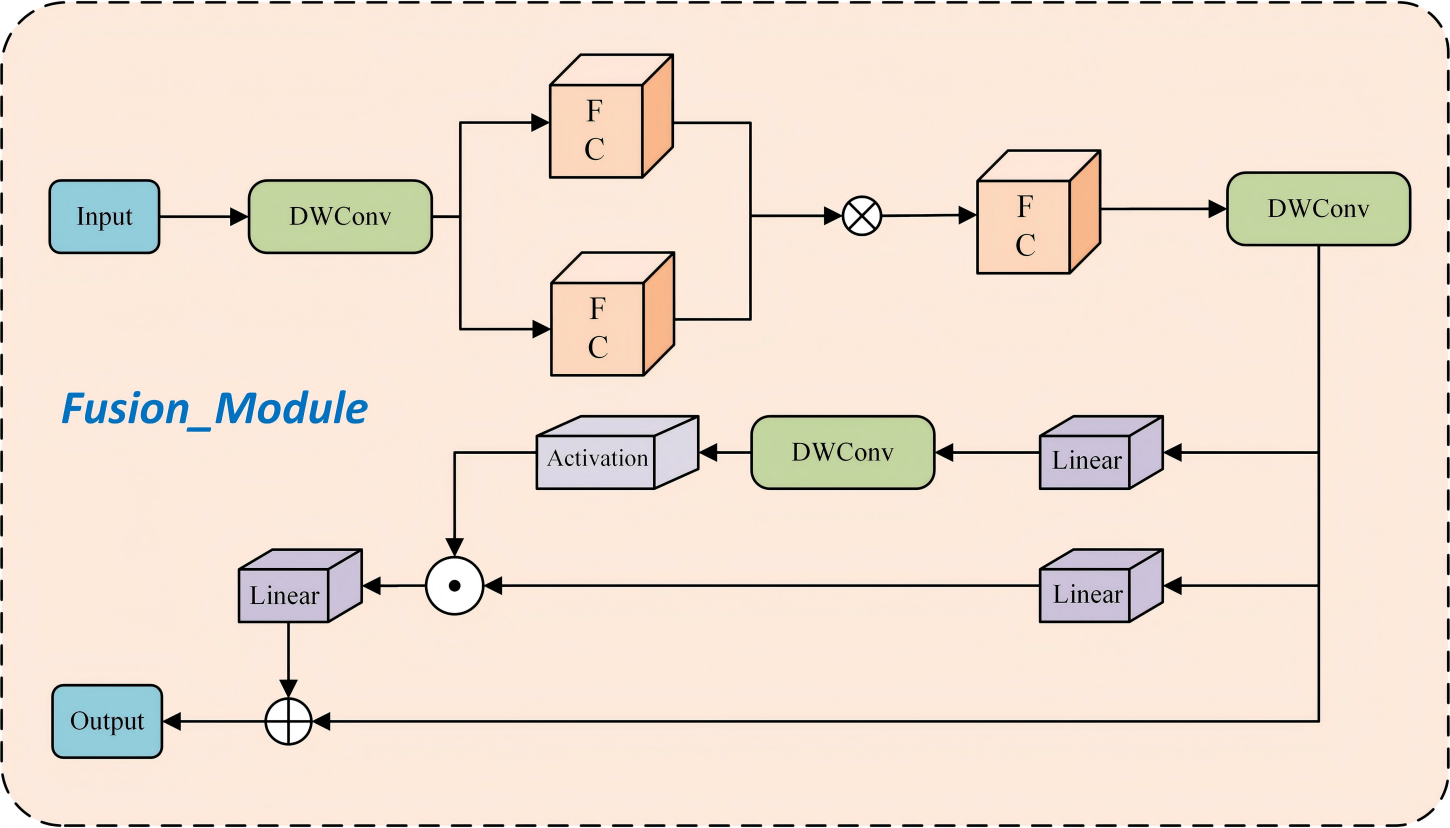
Structure of Fushion_Module.

To achieve network lightweighting while enhancing feature extraction capabilities, this study proposes the Fusion_Module. The first component of the Fusion_Module (**Figure 5**) is a simple yet effective network building block based on the “star operation” (element-wise multiplication). By performing element-wise multiplication (the star operation) within each layer, the input is mapped into a high-dimensional nonlinear feature space, conceptually similar to kernel methods. This design offers several key advantages: (1) High-dimensional feature mapping: The star operation transforms the input into an extremely high-dimensional nonlinear feature space without increasing network width. (2) Improved efficiency and model performance: Despite its structural simplicity, the module enhances the model’s learning capacity and generalization ability through high-dimensional feature transformation. (3) Computational efficiency: Compared to conventional summation operations, the star operation reduces computational overhead while maintaining superior performance.

The second component of the Fusion_Module structure (**Figure 5**) primarily consists of two linear projections that perform element-wise multiplication, one of which is activated by a gating function. A 3×3 depthwise convolution is introduced before the activation function in the gating branch to enhance feature sensitivity. This design leverages neighboring features to construct a gating-based channel attention mechanism. Unlike the global average pooling used in the SE mechanism (Niu et al., 2023), this component generates a unique gating signal for each token based on its local fine-grained features. The key advantage of this approach is that it enables each token to be assigned a distinct gating signal derived from its nearest fine-grained neighbors, effectively overcoming the limitation of the overly coarse representation associated with global average pooling.

**Figure 6:**
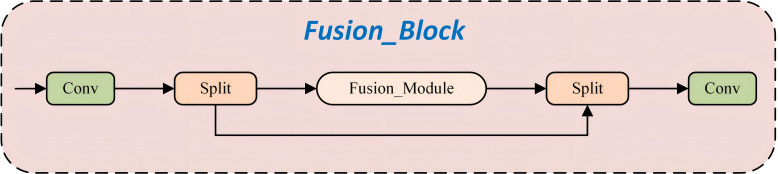
Structure of Fushion_Block.

The Fusion_Block (**Figure 6**) extracts and partitions input features at an early stage through convolutional operations and feature branching. One branch undergoes deep nonlinear processing and fine-grained gating via the Fusion_Module, while the other retains the original information. The two branches are subsequently fused through concatenation and integrated via a convolutional layer to produce the final output. This design facilitates efficient feature enhancement and multi-scale information interaction, thereby providing subsequent network layers with more accurate and comprehensive feature representations.

### 5.2 MS_Fusion

**Figure 7:**
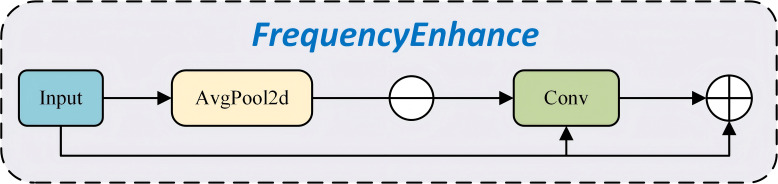
Structure of FrequencyEnhancer.

In this study, the frequencyEnhancer (**Figure 7**) is introduced to substantially enhance detailed information. This component enables the model to more effectively capture fine details and boundary features in the input images, thereby enhancing its sensitivity to subtle visual cues. The architecture of the frequencyEnhancer is structurally simple and computationally efficient. Moreover, nn.AvgPool2d is applied to blur the input feature maps through average pooling operations. This step extracts low-frequency components by smoothing the feature maps. Subsequently, enhanced edge (high-frequency) information is obtained by subtracting the blurred results from the original feature maps, leading to a more refined and accurate representation of image characteristics.

The edge or high-frequency components of the image are extracted by subtracting the blurred feature map from the original input feature map, thereby emphasizing fine details and contour structures. Next, a convolutional layer is applied to further refine the extracted edge information, aiming to enhance its representational capacity. Typically, activation functions such as Sigmoid are used to modulate the intensity of this enhancement process. The refined edge features are then integrated with the original input to generate an enhanced output. Finally, the processed edge information is added back to the original feature map to produce the final output. This integration effectively incorporates frequency-based edge features into the original representation, thereby improving the overall quality and expressiveness of the feature learning process.

**Figure 8:**
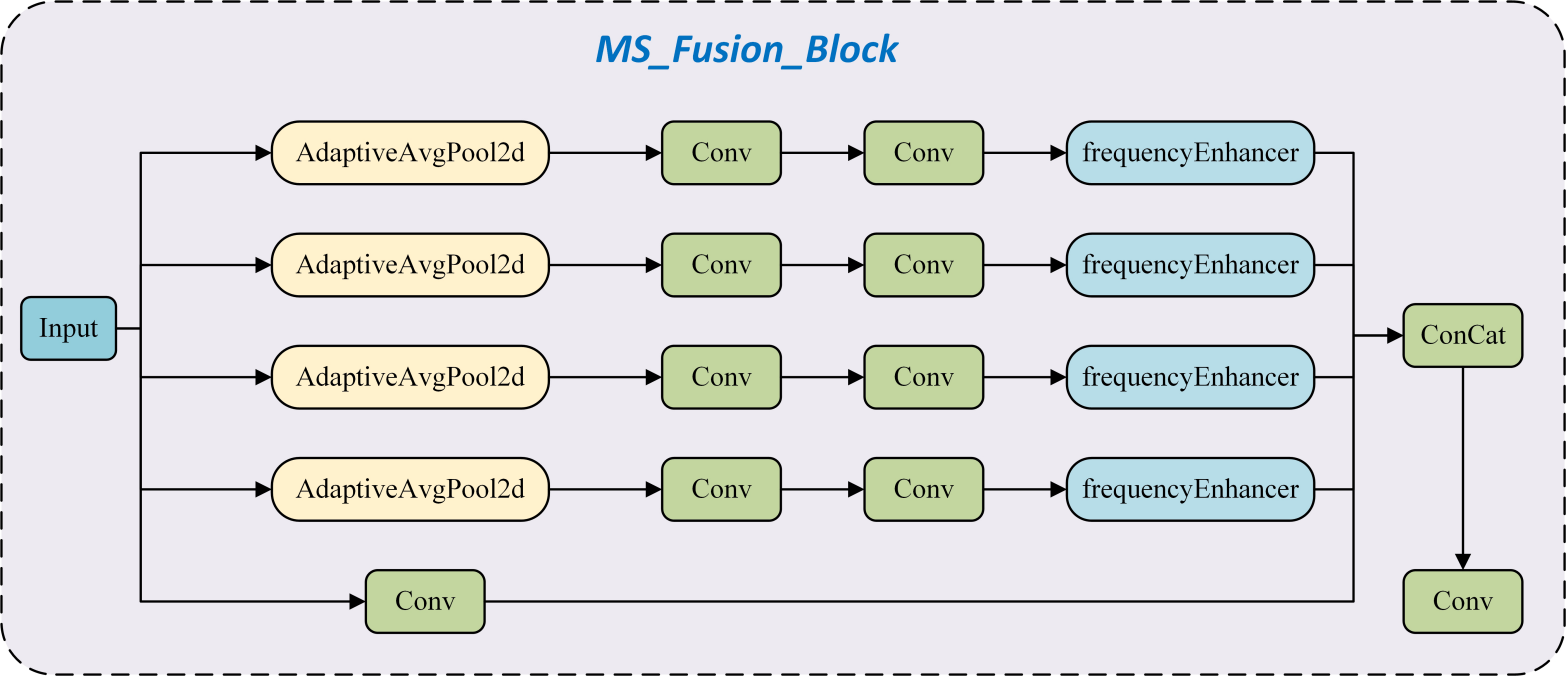
Structure of MS_Fusion_Block.

Building upon the frequencyEnhancer, this study proposes the MS_Fusion_Block architecture (**Figure 8**). For multi-scale feature extraction, nn.AdaptiveAvgPool2d is utilized to perform average pooling operations on the input feature maps across multiple scales. This strategy facilitates the acquisition of multi-level local information, enabling the model to effectively capture image features at varying resolutions and thereby improve its understanding of complex image structures. In terms of edge information enhancement, the proposed architecture incorporates the frequencyEnhancer module to focus on extracting and strengthening image edge features. By enhancing these edge features, the model achieves improved precision in processing fine details and boundaries. In the feature fusion stage, multi-scale features are rescaled to a uniform spatial dimension through interpolation techniques. This step ensures cross-scale compatibility and facilitates subsequent integration. The aligned features are then concatenated and further refined using convolutional layers to achieve effective feature fusion. This procedure generates a cohesive and enriched feature representation, thereby improving both the representational capacity of the features and the perceptual effectiveness of the model.

**Figure 9:**
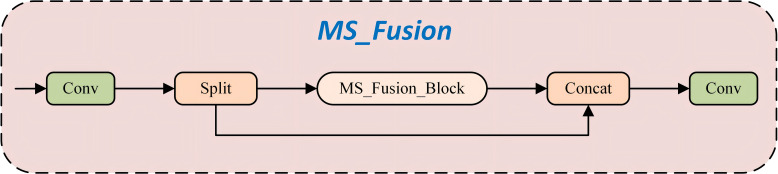
Structure of MS_Fusion.

Building upon the MS_Fusion_Block, this study proposes the MS_Fusion architecture (**Figure 9**), which integrates multiple MSEI modules. Through mechanisms of multi-scale feature extraction, edge information enhancement, and efficient feature fusion, the proposed architecture significantly improves the network’s capability in feature representation and overall performance. The MS_Fusion_Block maintains computational efficiency while effectively leveraging multi-scale and edge information to enhance the model’s performance across a wide range of image processing tasks. Its key functionalities are outlined as follows: (1) Multi-Scale Feature Extraction: Each MS_Fusion_Block module extracts features at different scales using adaptive pooling and convolutional layers, thereby capturing multi-level image information. This approach enables the model to understand image content at varying resolutions, thus enhancing its ability to perceive complex image structures. (2) Edge Information Enhancement: The MS_Fusion_Block incorporates the EdgeEnhancer module to focus on extracting and reinforcing edge-related features. Edge information is essential for identifying fine details and object boundaries, which contributes to improved performance in vision tasks such as object detection and semantic segmentation. (3) Feature Fusion and Optimization: In the MS_Fusion framework, outputs from multiple MS_Fusion_Block modules are fused to generate a more comprehensive and enriched feature representation. This strategy effectively integrates multi-scale and edge features, thereby strengthening feature expressiveness and boosting overall model performance. (4) Efficient Computation and Parameter Utilization: The architecture achieves high computational efficiency while making full use of model parameters. Through the parallel integration of multiple MS_Fusion_Block modules, it realizes both efficient and effective feature extraction and enhancement.

**Figure 10:**
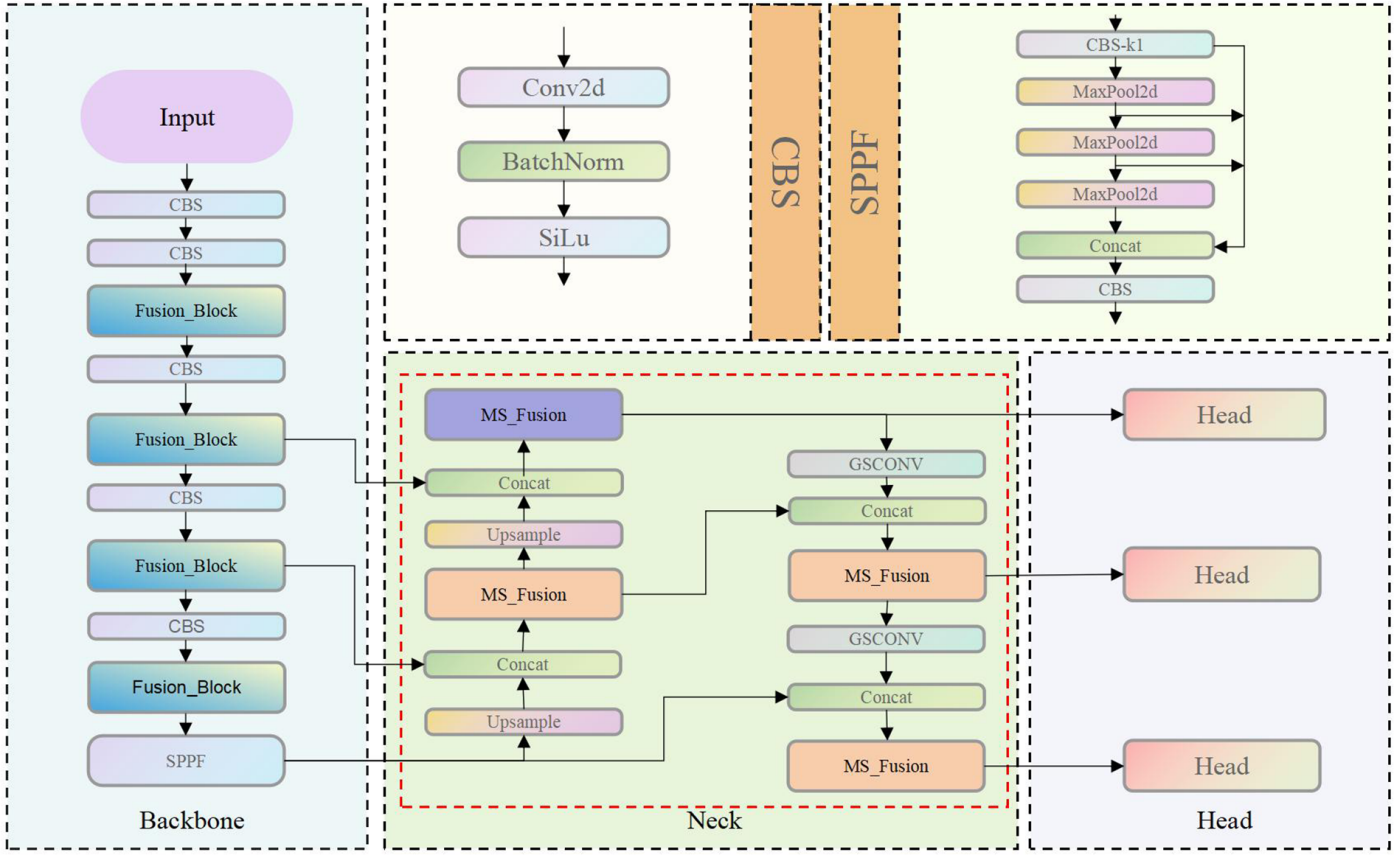
Structure of Yolo-Pika.

Building upon this foundation, this study proposes a novel neck network architecture termed MS_Fusion_FPN (**Figure 10**). This design innovatively integrates multi-scale feature extraction with frequency domain enhancement techniques, extending the conceptual framework of the traditional FPN. The architecture incorporates the MS_Fusion module, which extracts multi-scale features from feature maps at different hierarchical levels. This is achieved through adaptive average pooling and dual convolution operations. Following this, a dedicated frequencyEnhancer module is applied to enhance both high-frequency and low-frequency components, enabling a more precise and refined feature fusion process. Finally, by concatenating multi-channel features and applying integration convolution, MS_Fusion_FPN effectively fuses low-level detailed information with high-level semantic representations. This significantly improves the model’s ability to capture scale variations, edge details, and texture characteristics in object detection tasks, thereby enhancing overall detection accuracy and robustness.

By integrating the Fusion_Block and MS_Fusion_FPN into the YOLOv8 framework, this study develops the YOLO-Pika model. This model achieves superior performance in capturing both fine-grained details and global semantic information, while maintaining efficient inference speed.

## 6 Analysis of experimental results

### 6.1 Ablation experiments

To validate the effectiveness of the proposed YOLO-Pika network, this study conducted ablation experiments based on the YOLOv8n architecture. The comparative results are summarized in **Table 2**. When either the Fusion_Block or the MS_Fusion_FPN module was integrated into YOLOv8n, various model parameters were improved. Notably, the YOLO-Pika network, which combines both the Fusion_Block and MS_Fusion_FPN, demonstrates substantial performance gains across all evaluation metrics compared to the baseline and other variant configurations. Specifically, the parameter count is reduced by 22.67%, the FLOPs (floating-point operations per second) decrease by 0.01%, while maintaining an inference speed of 205 FPS. In conclusion, the proposed strategy of integrating multi-scale feature fusion and fine-grained attention mechanisms effectively enhances detection performance without introducing additional computational overhead or with only a marginal increase in computation.

**Table 2:** Ablation experiments.

Model	Fusion_Block	MS_Fusion_FPN	P	R	mAP50	mAP50-95	Parameter	Flops	FPS
YOLOv8n	×	×	85.2	82.1	90.0	48.2	3.00	8.1	200
YOLOv8n	✓	×	87.0	84.8	91.5	49.6	2.78	7.4	190
YOLOv8n	×	✓	87.3	86.4	92.4	51.2	2.44	8.2	213
YOLO-Pika	✓	✓	88.5	87.9	93.4	53.2	2.32	8.0	205

**Figure 11:**
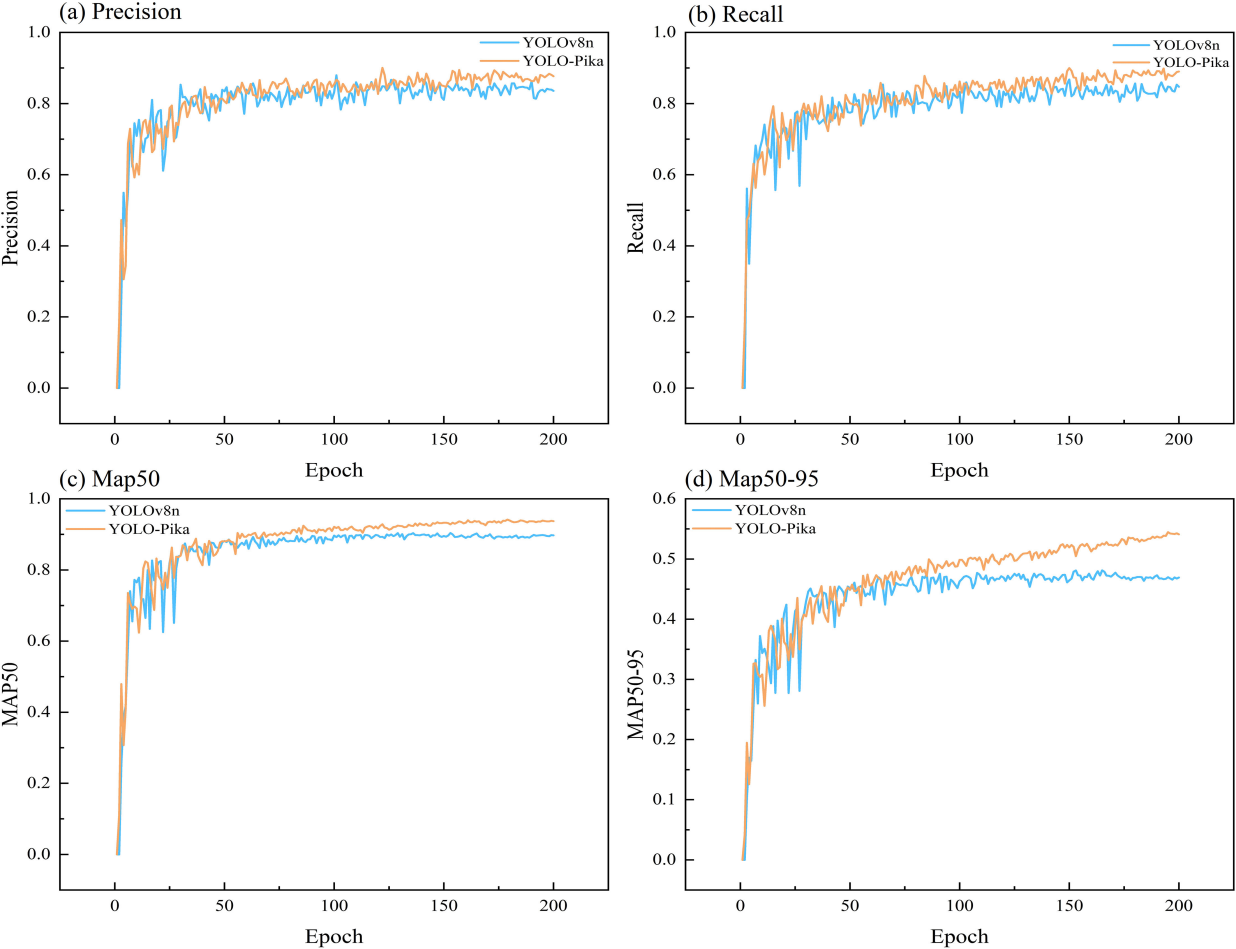
Analysis of the changing trends of different evaluation indicators for YOLO-Pika and YOLOv8 Networks.

A comparative analysis between the YOLOv8n and YOLO-Pika networks demonstrates that YOLO-Pika achieves superior performance in terms of precision (Equation 1), recall (Equation 2), mAP50, and mAP50-95. During the early training phase, the precision and recall of the YOLO-Pika model (**Figures 11a, b**) exhibited noticeable fluctuations. However, after approximately 100 epochs, these metrics stabilized and surpassed those of YOLOv8n. This suggests that YOLO-Pika achieves higher detection accuracy with fewer instances of missed detections. The comparison results for mAP50 and mAP50-95 (**Figures 11c, d**) further confirm the effectiveness of YOLO-Pika in detecting plateau pika burrows. The model demonstrates improved generalization capability and more accurate target localization, allowing it to better handle diverse detection scenarios and precisely identify target objects.

### 6.2 Comparative analysis of different models

In this study, a comparative analysis was conducted between YOLO-Pika and five models, including YOLOv8n (**Table 3**). The results clearly demonstrate that YOLO-Pika achieves notable improvements across all evaluation metrics compared to YOLOv8n. When compared with YOLOv5n and YOLOv6n, YOLO-Pika exhibits significant improvements in Precision, Recall, mAP50, and mAP50-95. Additionally, it involves fewer model parameters. However, it is important to note that the computational cost of YOLO-Pika is higher than that of YOLOv5n. In comparison with RT-DETR-l and YOLOv10n, YOLO-Pika shows consistent and substantial enhancements across all performance indicators. Based on this comprehensive evaluation, it can be concluded that YOLO-Pika delivers superior performance in the identification of plateau pika burrows.

**Table 3:** The comparative results of evaluation metrics for different models.

Model	Precision	Recall	mAP50	mAP50-95	Parameter	Flops
YOLOv5n	84.9	82.3	89.7	47.3	2.5	7.1
YOLOv6n	85.9	82.9	89.7	46.8	4.23	11.8
RT-DETR-l	81.5	81.3	87.7	45.6	31.99	103.4
YOLOv10n	83.7	80.2	88.9	47.0	2.70	8.2
YOLOv8n	85.2	82.1	90.0	48.2	3.00	8.1
YOLO-Pika	88.5	87.9	93.4	53.2	2.32	8.0

Regarding different target scales (specifically, the three scales of small targets (AP_S), medium targets (AP_M), and large targets (AP_L)) (Equation 3), the YOLO - Pika model exhibits performance improvements to varying extents compared to the YOLOv8n model (**Table 4**). More precisely, AP_S has increased significantly from 28.4% to 35%, representing an improvement of 6.6 percentage points. AP_M has risen from 50.3% to 55.4%, reflecting a gain of 5.1 percentage points. In addition, AP_L has improved from 58.7% to 60%. Notably, YOLO-Pika exhibits a particularly strong advantage in detecting small targets. These results indicate that the integration of Fusion_Block and MS_Fusion effectively enhances multi-scale target detection accuracy.

**Table 4:** The comparative results of the Average Precision (AP) metrics under different target scales.

Model	AP_S	AP_M	AP_L
YOLOv8n	28.4	50.3	58.7
YOLO-Pika	35.0	55.4	60.0

**Figure 12:**
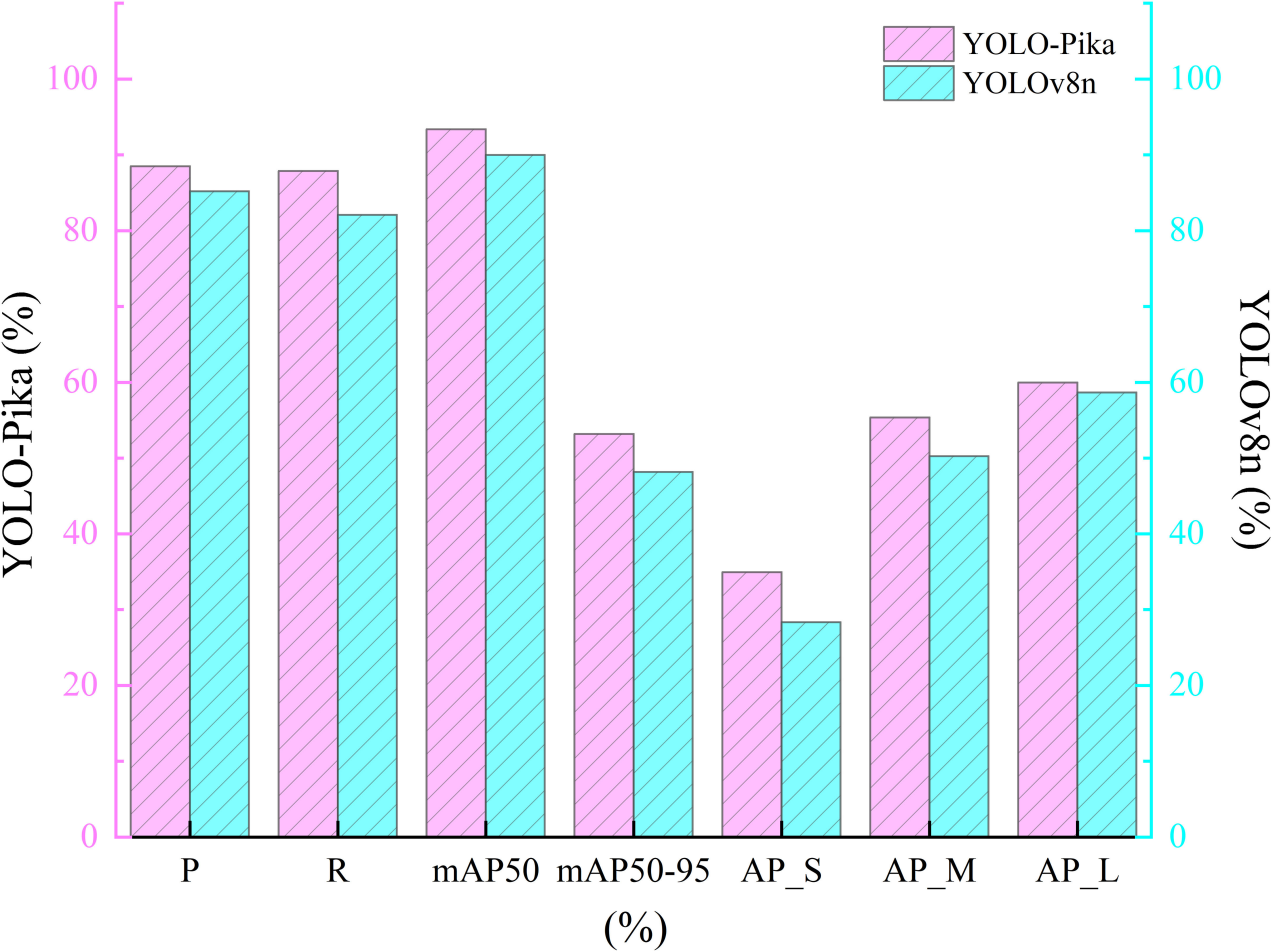
Comparison results of different evaluation metrics between YOLO-Pika and YOLOv8 networks.

In terms of precision, YOLO-Pika demonstrates a 3.3% improvement over YOLOv8n, indicating its superior performance in the accuracy of predicted bounding boxes. The recall rate of YOLO-Pika is also higher than that of YOLOv8n, with an increase of 5.8%. Under a relatively lenient IoU threshold (IoU = 0.5), YOLO-Pika achieves better detection precision. With respect to mAP50-95, YOLO-Pika shows a 5% improvement compared to YOLOv8. Overall, YOLO-Pika maintains high precision and recall while exhibiting enhanced adaptability to targets of varying shapes. These improvements suggest that the incorporation of Fusion_Block and MS_Fusion has significantly strengthened the model’s capability in feature representation and target detail capture (**Figure 12**).

### 6.3 Visualization experiment

**Figure 13:**
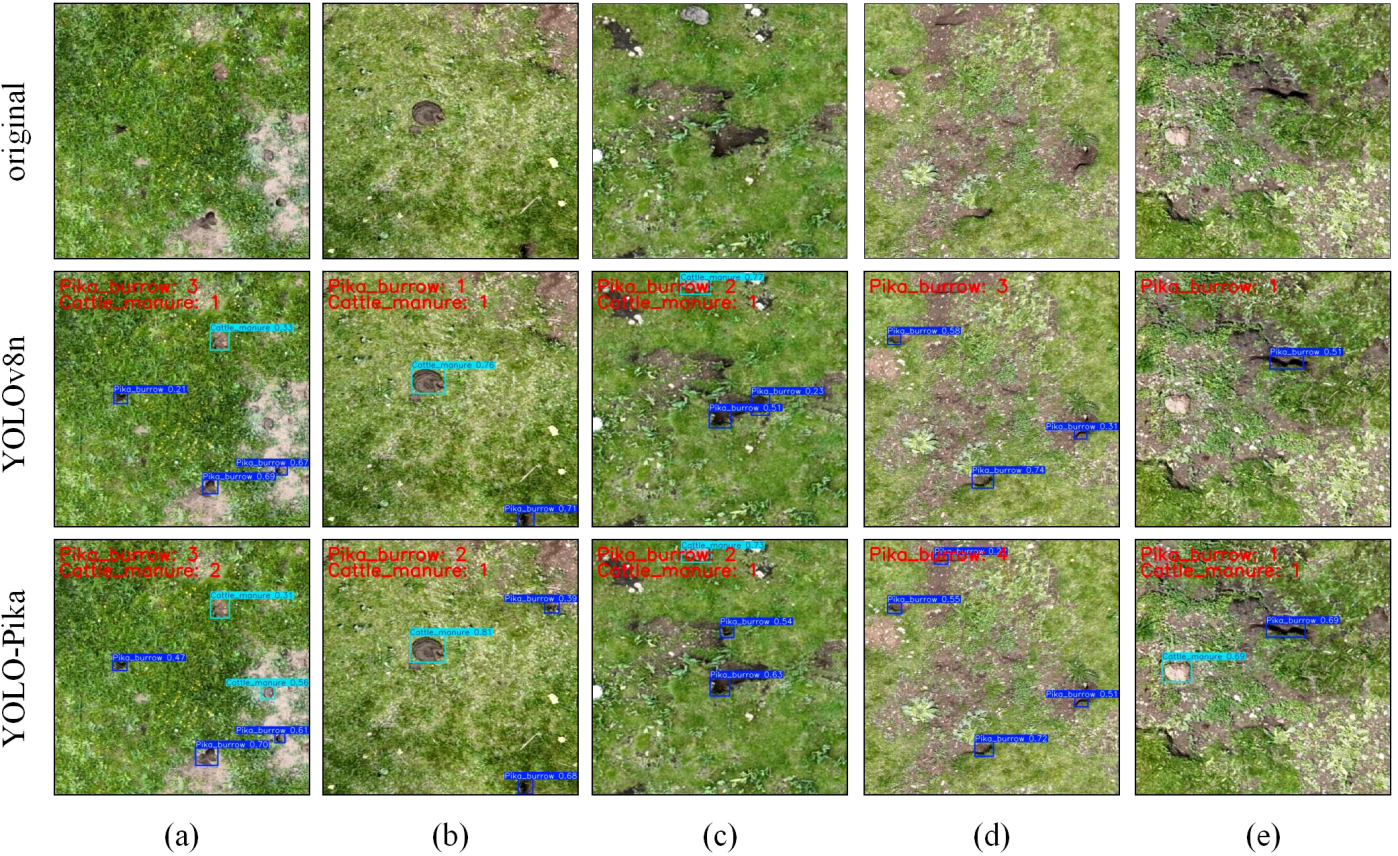
Visualization of results from YOLOv8n and YOLO-Pika on five sets of imagery: **(a–d)**, and **(e)**.

Qualitative analysis of five image groups (**Figure 13**) demonstrates that YOLO-Pika generally outperforms the baseline YOLOv8n in detecting pika burrows (highlighted by red bounding boxes) and cattle manure (highlighted by blue bounding boxes). Specifically, YOLO-Pika exhibits greater sensitivity to low-contrast or complex-textured scenes. It successfully detects burrows missed by YOLOv8n and reduces misclassification of stones and bare soil as cattle manure. In terms of detection confidence, YOLO-Pika surpasses YOLOv8n, with simultaneous reductions in both the missed detection rate and the false alarm rate. These observations indicate that the fine-grained gating mechanism introduced by Fusion_Block and the multi-scale frequency-domain enhancement in MS_Fusion_FPN collectively enhance the model’s capability in small-target discrimination and background suppression. Consequently, YOLO-Pika achieves more reliable and precise identification of pika burrows and cattle manure within complex alpine grassland environments.

**Figure 14:**
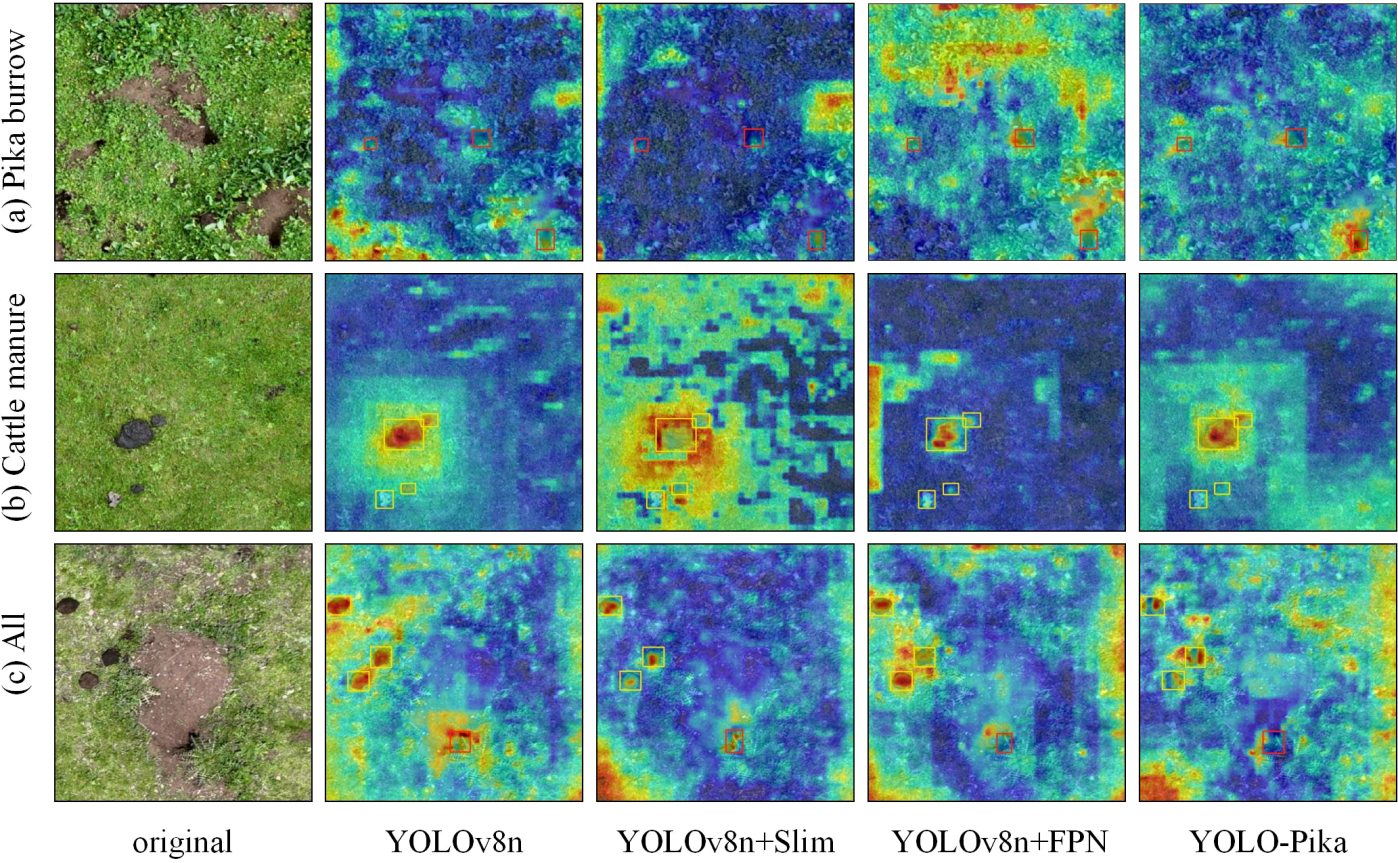
Heat maps before and after the embedding of the improvement modules (wherein YOLOv8n + Slim is the model with the Fusion_Block embedded and YOLOv8n + FPN is the model with the MS_Fusion_FPN embedded).

Furthermore, this study incorporated the Gradient-weighted Class Activation Mapping (Grad-CAM) technique into the model to visualize and analyze the feature extraction mechanisms of the network, generating corresponding heatmaps (**Figure 14**). This approach was employed to investigate how the enhanced modules influence the learning and representation of target features. In particular, for the detection of Pika burrows, YOLOv8n exhibited relatively scattered activation regions across the feature maps. In contrast, the YOLO-Pika model, which integrates the Fusion_Block and MS_Fusion_FPN modules, demonstrated a more concentrated and accurate focus around the entrances of the burrows. This result provides strong evidence that YOLO-Pika is more effective in capturing both the overall structure and detailed characteristics of Pika burrows. With regard to cattle manure detection, the heatmap generated by YOLOv8n displayed a certain degree of spatial diffusion, potentially leading to misclassification due to visual similarities with surrounding terrain features. In comparison, YOLO-Pika produced clearer and more localized activation zones within the primary target areas, indicating a higher level of discriminability and precision in its feature extraction process. In multi-target scenarios where various object categories coexist within the same image, YOLO-Pika demonstrated superior performance in generating more uniformly distributed and precisely localized attention hotspots across different target types, as compared to the other three models.

The results of the error rate indicators (**Table 5**) demonstrate that YOLO-Pika significantly outperforms YOLOv8n across six core error metrics. Specifically, the classification error (Ecls) decreased from 0.16 to 0.03, representing an 81.3% reduction. The localization error (Eloc) dropped from 3.23 to 1.70, a decrease of 47.4%. Furthermore, Eboth, which reflects combined classification and localization discrepancies, was reduced from 0.06 to 0.01, achieving a substantial 83.3% decline. In terms of redundancy and background interference, the duplicate detection error (Edupe) decreased from 0.22 to 0.18, while the background false-positive error (Ebkg) declined from 4.72 to 3.51. Additionally, the miss-detection rate (Emiss) was reduced from 0.64 to 0.33, corresponding to a 48.4% improvement. Collectively, these results indicate that YOLO-Pika, through the integration of Fusion_Block and MS_Fusion_FPN, not only enhances discriminative accuracy for challenging categories such as caves and cow dung but also effectively suppresses background noise and redundant bounding boxes. Particularly notable is the 40–80% error convergence achieved in localization and classification consistency for small targets, which strongly supports the model’s robustness and reliability in complex alpine grassland environments.

**Table 5:** Results of the error rate indicator for the plateau pika burrow dataset.

Model	Ecls	Eloc	EBoth	Edupe	Ebkg	Emiss
YOLOv8n	0.16	3.23	0.06	0.22	4.72	0.64
YOLO-Pika	0.03	1.70	0.01	0.18	3.51	0.33

### 6.4 Generalization experiment

To evaluate the generalization capability and scalability of YOLO-Pika across other publicly accessible rodent datasets, this study employed the Brandt’s vole hole dataset developed by (Wu et al., 2024), which utilized drone imagery. Brandt’s voles and plateau pikas are both classified as xeric burrowing rodents. The disturbances caused by their burrows (such as variations in hole diameter and bare soil patch characteristics) show high similarity in texture and spectral features. This similarity allows the dataset to serve as a valuable resource for transfer learning in cross-species burrow detection. Moreover, the dataset was collected from typical grasslands in Inner Mongolia, where the vegetation composition, surface background, and lighting conditions closely resemble those found in the alpine meadows on the northeastern margin of the Qinghai-Tibet Plateau. Therefore, it provides an effective benchmark for assessing the robustness of YOLO-Pika in handling diverse rodent burrow morphologies. It also enables the evaluation of the model’s practical applicability in similar grassland ecosystems, providing direct reference values for monitoring plateau pika burrows.

This study evaluated the performance of the YOLO-Pika model based on the dataset partitioning protocol proposed by (Wu et al., 2024), which divides the data into training, validation, and test sets at a ratio of 5:2:3 (**Table 6**). The results demonstrate that the two improved modules provide complementary benefits in enhancing model performance while managing resource consumption. Compared to the baseline YOLOv8n, incorporating only the Fusion_Block reduces computational load by 8%, while improving Precision by 6.7% and mAP50 (Equation 4) by 4.5%. When only the MS_Fusion_FPN is integrated, Recall increases by 8.8%, FPS rises to 213, and the number of parameters decreases by 19%, with FLOPs remaining nearly unchanged relative to the baseline. When both modules are combined to form the YOLO-Pika architecture, the model achieves optimal overall performance. Accuracy metrics are consistently improved, with parameter count and FLOPs reduced by 23% and 1%, respectively, compared to the baseline, while inference speed remains stable at 205 FPS. In conclusion, the Fusion_Block enhances fine-grained discrimination, and the MS_Fusion_FPN improves multi-scale feature fusion; these improvements complement each other effectively. These findings further confirm that YOLO-Pika offers strong generalization ability and lightweight efficiency for rodent burrow detection across diverse geographical environments.

**Table 6:** Ablation experiments on Brandt’s vole hole dataset.

Model	Fusion_Block	MS_Fusion_FPN	P	R	mAP50	Parameter	Flops	FPS
YOLOv8n	×	×	79.1	80.9	86.4	3.00	8.1	200
YOLOv8n	✓	×	85.8	84.7	90.9	2.78	7.4	190
YOLOv8n	×	✓	85.4	89.7	88.5	2.44	8.2	213
YOLO-Pika	✓	✓	87.1	88.6	91.3	2.32	8.0	205

In the error analysis conducted on Brandt’s vole hole dataset, both Ecls and Eboth were recorded as 0. This outcome stems from the fact that the dataset contains only a single category—”holes”—thereby precluding any classification errors or combined “classification + localization” errors. As a result, model performance is primarily evaluated through four key indicators: localization error, redundancy, background false positives, and missed detections. As shown in **Table 7**, YOLO-Pika consistently outperforms YOLOv8n across all critical error metrics. Specifically, the localization error (Eloc) decreases from 2.04 to 1.81, demonstrating that the multi-scale aggregation mechanism of MS_Fusion_FPN enhances bounding box regression accuracy. The duplicate detection error (Edupe) drops from 0.37 to 0.21, indicating that the fine-grained gating strategy within Fusion_Block effectively suppresses redundant predictions. The background false-positive error (Ebkg) is reduced from 4.44 to 3.98, highlighting the noise suppression capability of frequency-domain enhancement in complex grassland scenes. Finally, the miss-detection rate (Emiss) declines from 0.25 to 0.12, suggesting that the improved model exhibits greater sensitivity to small-scale or low-contrast holes. Overall, YOLO-Pika achieves a notable reduction in both localization and background-related errors, thereby providing strong empirical validation of the effectiveness of Fusion_Block and MS_Fusion_FPN in improving detection reliability and generalization performance.

**Table 7:** Results of the error rate indicator for the Brandt’s vole hole dataset.

Model	Ecls	Eloc	EBoth	Edupe	Ebkg	Emiss
YOLOv8n	0.00	2.04	0.00	0.37	4.44	0.25
YOLO-Pika	0.00	1.81	0.00	0.21	3.98	0.12

## 7 Discussion

### 7.1 Model performance analysis

YOLO-Pika has achieved stable and significant improvements in the accuracy-efficiency trade-off on two representative grassland burrow datasets. On the self-constructed plateau pika burrow dataset (**Table 2**), mAP50 increased from 88.9% to 92.3%, while mAP50-95 improved from 60.1% to 65.1%. At the same time, the number of model parameters was reduced from 3.00 M to 2.32 M, representing a 23% decrease. The computational cost, measured in FLOPs, remained approximately constant, and the real-time inference speed was maintained at 205 FPS. On the Brandt’s vole hole public dataset (**Table 6**), mAP50 further improved by 4.9 percentage points, with both Precision and Recall increasing by approximately 8 percentage points. Additionally, the model achieved the smallest parameter count and lowest computational load among compared methods.

In the plateau pika burrow dataset (**Table 4**), YOLO-Pika reduced classification, localization, and combined errors to 19%, 53%, and 17% of those observed in YOLOv8n, respectively. The rates of repeated detection, background false positives, and missed detections decreased by approximately 20-50%. These results demonstrate that Fusion_Block and MS_Fusion_FPN effectively reduce confusion between burrows and backgrounds, as well as between burrows and cow dung, thereby improving the detection performance for extremely small or occluded burrow entrances. For the single-category Brandt’s vole hole dataset (**Table 7**), where classification-related errors were inherently zero, YOLO-Pika still achieved reductions of 10-40% in localization errors, redundant detections, background false positives, and missed detections. This further confirms the cross-regional robustness of the proposed model.

Based on the Brandt’s vole hole dataset (Wu et al., 2024), reported the best performance of the lightweight YOLO series as follows: YOLOv5n achieved an mAP50 of 87.4% with 2.50 M parameters and 7.1 GFLOPs; YOLOv6n attained an mAP50 of 88.3% with 4.23 M parameters and 11.8 GFLOPs; YOLOv10n reached an mAP50 of 89.1% with 2.70 M parameters and 8.4 GFLOPs. The two-stage anchor-free detector FCOS achieved the highest accuracy for Brandt’s vole hole detection, with an overall mAP50 of 95.2%, at the cost of 32.1 M parameters and 32 GFLOPs. In contrast, the proposed YOLO-Pika model achieved an mAP50 of 91.3% under the same experimental conditions, using only 2.32 M parameters, 8.0 FLOPs, and achieving a real-time inference speed of 205 FPS. Compared to YOLOv5n, YOLOv6n, and YOLOv10-n, YOLO-Pika improved detection accuracy by 3.9, 3.0, and 2.2 percentage points, respectively, while maintaining the smallest model size. When compared to FCOS, YOLO-Pika demonstrated a slightly lower accuracy (3.9 percentage points), but significantly reduced the parameter count by approximately 93%, decreased computational complexity by about 75%, and increased inference speed nearly fourfold.

These results demonstrate that the integration of Fusion_Block and MS_Fusion_FPN achieves a more favorable trade-off between detection accuracy, model complexity, and inference efficiency. Consequently, YOLO-Pika offers substantial advantages in terms of deployment efficiency and generalization capability for resource-constrained UAV-based grassland monitoring applications.

### 7.2 Uncertainty analysis

During field investigations, environmental elements such as grasslands and rock shadows frequently appear in UAV imagery, often leading to false detections by YOLO-Pika. To address this challenge, this study synthesized the experimental protocols proposed by (Du et al., 2022) and (Wu et al., 2024). The UAV was flown at an altitude of 15 meters, and all field operations were conducted between 8:00 a.m. and 5:00 p.m. Data acquisition was systematically performed within this timeframe. This strategy ensured scientific rigor while effectively reducing the impact of various noise sources on model recognition performance, thereby improving the reliability and accuracy of the experimental outcomes.

Although YOLO-Pika has significantly improved the detection performance of plateau pika burrows under lightweight constraints through the integration of Fusion_Block and MS_Fusion_FPN, the current version deliberately omits explicit attention mechanisms to avoid increasing model parameters and inference latency. A considerable body of literature (Hu et al., 2018; Wang et al., 2020b; Yang et al., 2021) demonstrates that lightweight attention mechanisms—such as Squeeze-and-Excitation, ECA (Efficient Channel Attention), CBAM (Convolutional Block Attention Module), Coordinate Attention, and SimAM—are effective in enhancing channel dependencies and capturing long-range spatial relationships. Notably, these mechanisms offer substantial improvements in feature representation for extremely small or densely distributed targets. Therefore, the absence of such attention modules may constitute a key limiting factor in further improving the precision and recall of YOLO-Pika. To address this limitation, future work will investigate “plug-and-play” lightweight attention architectures under strict constraints, aiming to maintain increases in both parameter count and inference latency below 1%.

Despite demonstrating stable performance across two datasets, the model has not yet been validated in a broader range of ecological environments, such as alpine shrublands and desert meadows, nor has it been tested across diverse rodent species, including marmots. Moreover, due to the top-down imaging perspective of the UAV, YOLO-Pika may fail to detect plateau pika burrows that are partially or fully occluded by surrounding objects. Therefore, future research will focus on conducting real-time, large-scale detection of plateau pika burrows. This effort aims to investigate the impacts of both natural and anthropogenic factors on the spatial distribution and behavioral patterns of plateau pikas. These insights will contribute to the development of more precise and actionable recommendations for rodent control and the conservation of grassland ecosystems.

## 8 Conclusion

This study is based on a self-constructed dataset of plateau pika burrows and the publicly available Brandt’s vole hole dataset. It introduces and evaluates YOLO-Pika, an efficient detection model specifically designed for grassland rodent burrows. Compared with YOLOv8n, YOLO-Pika demonstrates several key improvements: (a) The C2f module in the backbone network is replaced by Fusion_Block. Through high-dimensional mapping and fine-grained gating mechanisms, this change reduces model parameters by 23% while minimizing redundant convolutional operations, all without compromising detection accuracy. (b) A multi-scale fusion feature pyramid network (MS_Fusion_FPN) is introduced in the neck architecture. This component effectively integrates frequency-domain information across multiple scales, thereby enhancing the semantic representation of small-aperture burrows. (c) The upsampling pathway and cross-layer connections have been simplified, resulting in a 1% reduction in FLOPs, while maintaining an inference speed of 205 FPS. (d) Without requiring additional detection heads, YOLO-Pika achieves a significant improvement in the recall rate for small targets. The lightweight design of the model is further highlighted by its compact file size of only 7.8 MB, making it highly suitable for deployment on embedded UAV platforms. Experimental results show that on the plateau pika dataset, YOLO-Pika improves mAP50 by 3.4 percentage points, mAP50-95 by 5 percentage points, and AP_S by 6.6 percentage points. On the Brandt’s dataset, mAP50 increases by 4.9 percentage points, while the average false detection rates related to localization errors, redundancy, and background clutter are reduced by 30-50%. When benchmarked against five mainstream lightweight object detectors, YOLO-Pika achieves the highest detection accuracy despite having the fewest parameters. In conclusion, YOLO-Pika effectively combines real-time responsiveness, detection precision, and deployment feasibility, offering a practical and cost-effective solution for large-scale monitoring of grassland rodent populations.

Looking forward, future research will explore the integration of lightweight attention modules such as ECA and SimAM to further enhance the recall performance for extremely small targets. Additionally, edge inference efficiency will be improved through techniques like INT8 quantization and pruning, aiming to meet the real-time ecological monitoring requirements of low-power UAV platforms.

## Data Availability

The raw data supporting the conclusions of this article will be made available by the authors, without undue reservation.
